# Activation of vascular endothelial cells by synovial fibrosis promotes Netrin‐1‐induced sensory nerve sprouting and exacerbates pain sensitivity

**DOI:** 10.1111/jcmm.17950

**Published:** 2023-09-13

**Authors:** Zhenyuan Ma, Yibao Wei, Taiyang Liao, Lishi Jie, Nan Yang, Likai Yu, Peimin Wang

**Affiliations:** ^1^ Department of Orthopaedics and Traumatology Affiliated Hospital of Nanjing University of Chinese Medicine, Nanjing University of Chinese Medicine Nanjing China; ^2^ Key Laboratory for Metabolic Diseases in Chinese Medicine First College of Clinical Medicine, Nanjing University of Chinese Medicine Nanjing China; ^3^ Jiangsu Province Hospital of Chinese Medicine Nanjing China; ^4^ Jiangsu Provincial Engineering Research Center of TCM External Medication Development and Application Nanjing China

**Keywords:** endothelial cell activation, nerve sprouting, Netrin‐1, peripheral pain sensitivity, synovial fibrosis

## Abstract

Synovial fibrosis is one of the most dominant histopathological changes in osteoarthritis of the knee (KOA), and activation of vascular endothelial cells in synovial fibrosis is both an important factor in mediating pain in KOA and a major contributor to the generation of pain signals. At the same time, angiogenesis and nerve fibres are more likely to underlie the pathology of pain induced by synovial fibrosis. In the present study, we established a co‐culture model of human umbilical vein endothelial cells (HUVECs) with dorsal root ganglion (DRG) and detected tissue and cellular Netrin‐1, vascular cell adhesion molecule‐1 (VCAM‐1), intercellular cell adhesion molecule‐1 (ICAM‐1), growth‐associated protein‐43 (GAP43), colorectal cancer deleted (DCC), uncoordinated 5 (UNC5), and the related expression of calcitonin gene‐related peptide (CGRP), substance P (SP) and nerve growth factor (NGF) in supernatant by ELISA to investigate the intervention of vascular endothelial cell activation on sensory nerve sprouting exacerbating peripheral pain sensitivity and to investigate the effect of Netrin‐1 from the perspective of Netrin‐1 secretion to illustrate its effector mechanism.

## 1 INTRODUCTION

Osteoarthritis of the knee (KOA) is a chronic degenerative disease of the knee with pain as the primary clinical symptom, and is widespread in the middle‐aged and elderly populations. For many years, guidelines have consistently recommended pain control as an important main line of treatment for KOA. Guidelines of 2019 published by the Osteoarthritis Research Society International (OARSI) clearly states that pain is recommended to be targeted as an independent pathological factor in KOA.^1^, ^2^, ^3^ Therefore, correcting peripheral pain sensitivity is important to alleviate KOA pain to improve the quality of life in KOA patients. There is a large series of research suggesting that synovial fibrosis is an important player in the development of KOA pain. For example, Nanus et al. showed that synovial tissue at the site of joint pain in KOA patients exhibited a differential phenotype with different fibroblast subpopulations, suggesting a close association between synovial fibrosis and KOA pain.^4^ Also, in our previous study, we showed that the degree of synovial fibrosis in KOA was positively correlated with pain sensitivity in KOA model rats.^5^

In KOA synovial fibrosis, cell activation, including synovial cells and vascular endothelial cells, is an important pathological mechanism.^6^ Vascular endothelial cell activation not only promotes angiogenesis, but also constitutes an important factor mediating KOA pain, and the secretion of fibroblast growth factor and epidermal growth factor may also be directly responsible for pain signals.^7^ Thus, areas of synovial fibrosis in KOA are often accompanied by vascular opacification and marked angiogenesis is highly similar to rheumatoid arthritis,^8^, ^9^, ^10^, ^11^ and angiogenesis and nerve fibres are more likely to be the anatomical basis for pain induced by synovial fibrosis. Studies have shown that intercellular cell adhesion molecule‐1 (ICAM‐1), vascular cell adhesion molecule 1 (VCAM‐1) are important indicators of vascular endothelial cell activation and play a key role in chronic inflammatory diseases.^12^ Previous studies have shown that peripheral pain sensitivity is closely related to the number and density of sensory nerve fibres and the way they innervate injury receptors.^13^ Further studies have shown that damaged sensory nerve fibres will rapidly sprout and grow, altering the original stimulus perception and intensity of information transmission.^14^

Among the important factors that enable neural sprouting, axon guidance factors play an important role. Among the family of axon guidance factors, the most studied one is the autocrine soluble protein Netrin‐1, a member of the laminin‐like protein family, which was originally identified as a potent chemotactic molecule involved in axon guidance and cell migration during embryonic development.^15^, ^16^ Numerous studies have shown that Netrin‐1 is extensively involved in the regulation of angiogenesis, inflammation, tissue remodelling and cancer, and that Netrin‐1 achieves directional regulation of sensory nerve sprouting through binding to the neuronal surface receptor DCC/UNC5.^17^ Netrin‐1/DCC has an attractive effect on neuronal axons, directing the growth of nerve fibres towards the damaged site, possibly by altering the density of nerve fibres to influence the strength of the message transmission; whereas Netrin‐1/UNC5 exhibits more of a repulsive effect, i.e. hindering the growth of nerve fibres towards the damaged site, which also indicates the significant research value of Netrin‐1 in the context of KOA pain.^18^, ^19^, ^20^, ^21^ Recent studies indicate that Netrin‐1‐mediated neurodirected sprouting is involved in peripheral pain sensitization, as in the study by Li et al. who found that electroacupuncture treatment relieves neuropathic pain by activating opioid receptors, decreasing DCC and Netrin‐1 expression and increasing UNC5h2 expression in the dorsal horn of the spinal cord.^22^ It has also been observed that KOA osteoclasts secrete Netrin‐1, which mediates mechanical nociception in KOA mice through DCC receptors that are attractively directed to dorsal root ganglion DRG neurons.^23^

We therefore speculated that the stimulation of endothelial cell activation by synovial fibrosis may promote the secretion of the axon guidance factor Netrin‐1, which drives sensory nerve sprouting leading to peripheral pain sensitivity.

## 2 MATERIALS AND METHODS

### 2.1 Reagents

The primers were supplied by Sangon Biotech. The enzyme‐linked immunosorbent assay (ELISA) kits for CGRP, SP and NGF were supplied by Invitrogen (Life Technologies Corp.). Fetal bovine serum (FBS), bovine serum albumin (BSA), Dulbecco's Modified Eagle's Medium (DMEM), TRIzol, and 0.25% trypsin‐ethylenediaminetetraacetic acid (trypsin–EDTA) were purchased from Gibco (Life Technologies Corp.). Cell Counting Kit‐8 (CCK‐8) was purchased from Dojindo. Antibodies: Netrin‐1 (DF8579, affinity), VCAM‐1 (DF6082, affinity), ICAM‐1 (16174‐1‐AP, proteintech), GAP43 (8945s, Cell Signaling Technology), DCC (DF3611, affinity) and UNC5 (DF13685, affinity). Monoidoacetate acid, type I collagenase and dimethylsulfoxide (DMSO) were all obtained from Sigma. All other chemicals were of reagent grade. Goat anti‐rabbit IgG H&L (HRP) and Picro Sirius Red Stain kit were also supplied by Abcam.

### 2.2 Rat model and experimental design

Fifty male SD rats, 4–5 weeks old, weighing 220–260 g (provided by Beijing Vitong Lihua Animal Technology Co., Ltd.), were housed under standard conditions: temperature 21 ± 1°C, relative humidity 50%–80%, and a 12:12 h light dark cycle. All animals underwent experiments in accordance with the National Institute of Health Guide for the care and use of animals. All male SD rats were randomly divided into five groups: Normal group (*n* = 10), KOA group (*n* = 10), TGF group (*n* = 10), TGF inhibitor group (*n* = 10), and Netrin‐1 group (*n* = 10). The KOA model was constructed by ACLT surgery in all groups except the normal group, and the successful model was verified by drawer test. Our previous data showed that at Day 14, knee diameter was significantly larger than control. After successful moulding, the TGF group, TGF inhibitor and Netrin‐1 group were injected intraperitoneally at doses of 200 ng/50 μL, 5 mg/kg/day and 500 pmol, respectively according to the instructions for aggravating synovial fibrosis with TGF‐β recombinant protein, TGF‐β inhibitor and Netrin‐1 antibody (all injections were dissolved in 0.9% saline). On Day 28 of the intervention (after the last dose), all rats were anaesthetised and blood was collected from the abdominal aorta, and synovial tissue from the knee and DRG tissue from L3 to L5 of the spine were collected for the next step of the experiment. All animals are provided by Beijing Viton Lihua Laboratory Animal Technology Co Ltd (Animal Production License No. SCXK (Beijing) 2021‐0011) and all animals are housed at the Laboratory Animal Centre of Nanjing University of Chinese Medicine (Animal Ethics No. 202208A032).

### 2.3 Cell culture

HUVECs were purchased from American Type Culture Collection (ATCC). HUVEC conducted subsequent experiments after passing to the third generation using endothelial cell specific culture medium (ECSM, Zhongqiao Xinzhou, ZQ‐1304). DRG: Trypsinized dorsal root ganglia from L3 to L5 were prepared as single‐cell suspensions in DMEM medium (containing 1% FBS), seeded in small dishes, incubated in a 37°C incubator for 6 h and then cultured with DMEM/F12 + 2% B27. During the cell intervention, the LPS, TGF, TGF inhibitor and Netrin‐1 groups were treated with 5 μg/mL of LPS, while the TGF, TGF inhibitor and Netrin‐1 groups were treated with 10 ng/mL, 10 μmol and 200 pmol, respectively, according to the instructions for the subsequent experiments.

### 2.4 Western blotting (WB)

Synovial tissues, DRG issue, HUVEC and DRG cell were mixed with radioimmunoprecipitation assay (RIPA) lysate and grinded for 10–15 min. The samples were agitated on ice for 30 min and the supernatant was collected. The protein levels were quantified with a bicinchoninic acid (BCA) protein assay kit (Roche, Basel, Switzerland). Then, the protein samples were electrophoresed in sodium dodecylsulphate polyacrylamide gel electrophoresis (SDS‐PAGE) to separate the proteins. Proteins were transferred from gel onto a polyvinylidene fluoride (PVDF) membrane and blocked with 5% nonfat milk for 2 h. The membrane was incubated with the primary antibody (1:1000) at 4°C overnight and then with the second antibody for 2 h. Bands were visualized via exposure to the electrochemiluminescence (ECL) method, and the overallgray value of protein bands (average gray value area) was quantified. Glyceraldehyde‐3‐phosphate dehydrogenase (GAPDH) was used as the internal marker. The relative protein expression was taken as the target protein gray value/internal reference gray value.

### 2.5 Real‐time reverse transcription‐polymerase chain reaction (qRT‐PCR)

RNA was isolated from tissue and cells with Trizol (Invitrogen). Reverse transcription was performed using a first‐strand cDNA synthesis kit (Takara) according to the manufacturer's instructions. Quantitative polymerase chain reaction (qPCR) was performed using Premix Ex Taq SYBR‐Green PCR (Takara) according to the manufacturer's instructions on an ABI PRISM 7300 device (Applied Biosystems). The primer was designed and synthesized by the Shanghai Biotechnology Service Company. Primers Sequences were as follows:

**Table:** 

Name	Primer sequences
Netrin‐1	F:5′‐AAGCCCTTCCACTACGAT‐3′
	R:5′‐GTTGAGACAGACACCCCC‐3′
VCAM1	F:5′‐AGGGATTAATGAGGCTGG‐3′
	R:5′‐TTGACGCTCTTAGATGGG‐3′
ICAM1	F:5′‐TACCTACAAGTGCCGTGC‐3′
	R:5′‐CCAACAATTATGACCCAG‐3′
GAP43	F:5′‐CGTTGCTGATGGTGTGGA‐3′
	R:5′‐CTTGGAGGACGGCGAGTT‐3′
DCC	F:5′‐ATCCTCCCGTCTCCCACA‐3′
	R:5′‐TTCCTGCTCCGAAACCTC‐3′
UNC5	F:5′‐GAGGGCAGTTGGGAGGAT‐3′
	R:5′‐ACGGGAGCAAACAGAAGG‐3′
β‐Actin	F:5′‐GAGAGGGAAATCGTGCGT‐3′
	R:5′‐GGAGGAAGAGGATGCGG‐3′

The PCR reactions as follow: per well: 0.4 μL of forward and reverse primers, 10 μL 2 × ChamQ SYBR qPCR Master Mix, 1 μL CDNA, and 8.2 μL ddH2O; three replicate wells were performed using an ABI 7500 qRT‐PCR system (Applied Biosystems). The following reaction conditions were employed: 95°C for 30 s, 95°C for 10 s and 60°C for 30 s; the third stage (melting curve), 95°C for 15 s, 60°C for 60 s and 95°C for 15 s. The relative expression of mRNA was adjusted using GAPDH as the internal reference and calculated using the method of 2^−ΔΔCt^.

### 2.6 Staining of pathological tissue sections

Rat synovial tissue was fixed with 4% paraformaldehyde, embedded in paraffin, sectioned, stained according to Sirius red staining, HE staining instructions and Masson staining, and tissue changes were observed under light microscopy.

### 2.7 Silver staining of nerves

Tissues were removed from 4% paraformaldehyde, first paraffin sectioned and deparaffinized with xylene and absolute ethanol, and then invaded into the acid formaldehyde dye. After washing, sections were immersed in glycine silver dye. Finally, water sealed sections were stained with reducing solution and images were acquired using a Nikon eclipsee100 (Nikon).

### 2.8 Immunohistochemistry

Immunohistochemical staining of synovial tissue and DRG tissue. According to the instructions of the immunohistochemical kit, paraffin sections were successively dewaxed to water, antigen repair, endogenous peroxidase blocking, blocking, primary and secondary antibody incubation, colour rendering, restaining of the nucleus, dehydration and sealing, and microscopic observation.

### 2.9 Immunofluorescence

Remove synovial tissue and DRG tissue from 4% paraformaldehyde, follow the immunofluorescence staining instructions for embedding, dewaxing, sectioning, antigen repair, endogenous enzyme blocking, blocking, primary and secondary antibody incubation, followed by incubation with TSA reagent and PBS cleaning three times, repeated primary and secondary antibody incubation and TSA reagent incubation, followed by PBS cleaning three times, DAPI staining, and microscopic observation. Endothelial cells and DRG cells were first washed with PBS three times, each time for 3 min, then fixed with 4% paraformaldehyde PBS for 5 min, and then sealed with 1% BSA for 1 h. Then incubate the cells with primary and secondary antibodies according to the instructions of the kit and observe under a microscope.

### 2.10 Enzyme‐Linked Immunosorbent Assay (ELISA)

CGRP, SP and NGF levels in the culture media were determined using a commercially available rat ELISA kit according to the manufacturer's instructions. The rat peripheral serum and cell culture supernatants were collected and centrifuged at 10,000 rpm for 20 min at 4°C.

### 2.11 Paw withdrawal experiments on a cold plate

On Days 0, 14, 28 and 42, all rats were placed on a cold glass surface to determine their paw retraction time. All rats were briefly placed on a temperature adjustable cold plate (0 ± 1°C, 35150‐001, Ugo Basil SLR), with transparent cylindrical plastic plates placed on the cold plate and covered with perforated transparent pressure plates. We recorded the time from the beginning until the limb left the glass panel.

### 2.12 Statistical analyses

The statistical analysis was performed using the SPSS 20.0 software (SPSS Inc., Chicago). Data are presented as the mean ± standard deviation. Group comparisons were assessed with one‐way anova or two‐way anova with Bonferroni's post hoc test for comparison of multiple columns. A value of *p* < 0.05 (two‐tailed) was considered statistically significant.

## 3 RESULTS

### 3.1 KOA synovial fibrosis activates vascular endothelial cells to promote Netrin‐1 secretion

**FIGURE 1:**
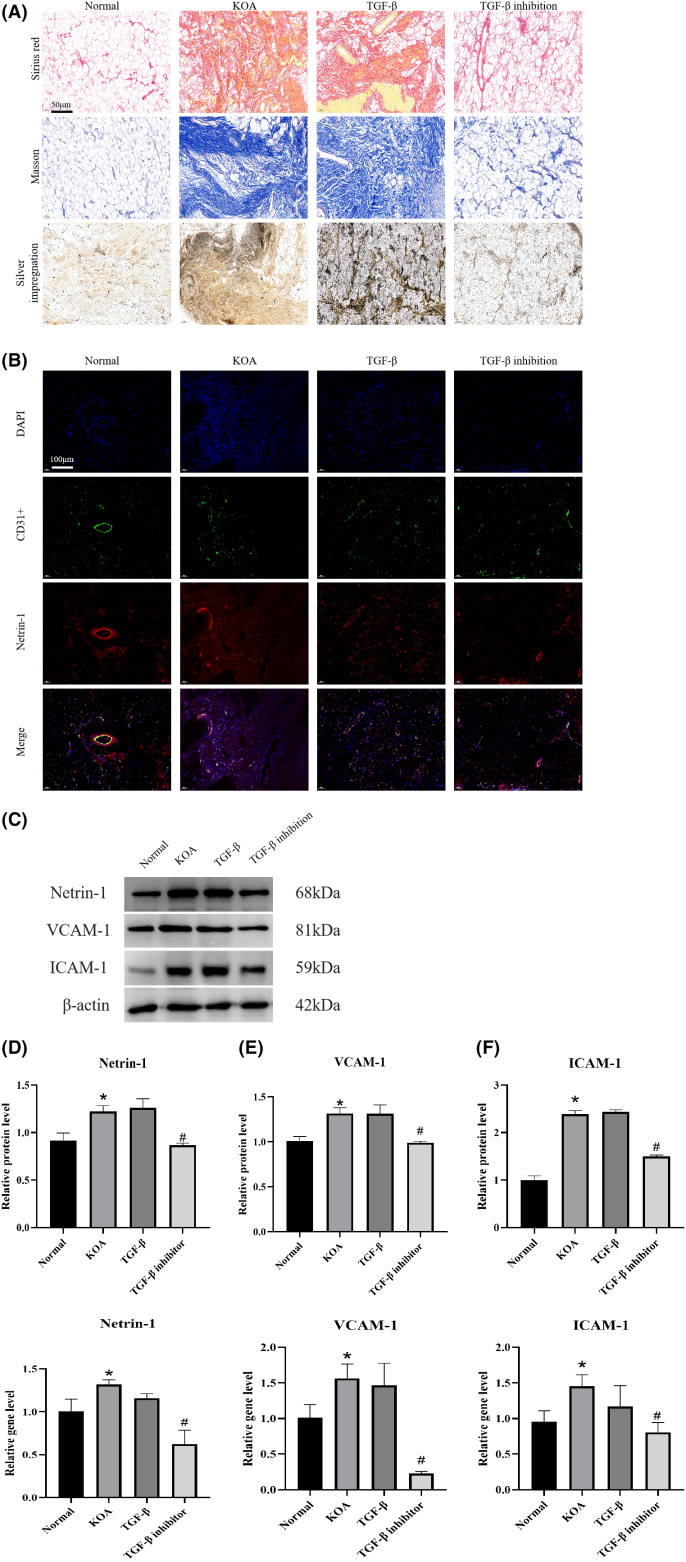
KOA synovial fibrosis activates vascular endothelial cells to promote Netrin‐1 secretion. (A) Each group was stained with Sirius Red stain and Masson stain for rat synovial tissue, 200× scale bar = 50 μm, showing collagen fibre content. Each group was stained with synovial tissue using silver plated nerve, 200x scale bar = 50 μm, showing neuronal and nerve fibre content. (B) Each group used Netrin‐1 + CD31 immunofluorescence staining of synovial tissue, 100× scale bar = 100 μm, to show the expression of the vascular endothelial cell marker CD31 and the expression of Netrin‐1 in synovial tissue. (C–E) WB and PCR were performed to detect the protein and gene expression of Netrin‐1 and vascular endothelial cell activation markers ICAM‐1 and VCAM‐1 in the synovial tissues of rats in each group. (*p < 0.05, *^#^p <* 0.05).

In order to investigate the induction of vascular endothelial cell activation and Netrin‐1 secretion by KOA synovial fibrosis, synovial tissues from Normal, KOA, TGF‐β and TGF‐β inhibition groups were stained with Sirius red, Masson, nerve silver plating and immunofluorescence staining, respectively. The expression of genes and proteins of vascular endothelial cell activation index and nerve sprouting index in synovial tissues were measured by WB and qPCR. As shown in Figure 1A, compared with the Normal group, the expression of collagen fibres in the synovial tissues of the KOA and TGF‐β groups was significantly increased and fibrosis was severe, and the expression of neurons and nerve fibres was also significantly increased, while the expression of those markers in the TGF‐β group was more than that of the KOA group. In the TGF‐β inhibition group, the expression of collagen fibres, neurons and nerve fibres was significantly reduced compared to the KOA and TGF‐β groups, suggesting a decrease in nerve sprouting. As shown in Figure 1B, immunofluorescence staining of Netrin‐1 and CD31 in the synovial tissues of each group showed that the expression of the vascular endothelial cell marker CD31 was significantly increased in the KOA and TGF‐β groups compared to the Normal group and the expression regions of Netrin‐1 and CD31 were highly overlapping, while in the TGF‐β inhibition group, the expression of both CD31 and Netrin‐1 was significantly reduced in the TGF‐β inhibition group. As shown in Figure 1C–F, the protein and gene expression of Netrin‐1, an indicator of nerve sprouting, and ICAM‐1 and VCAM‐1, indicators of endothelial cell activation, were significantly increased in synovial tissue in the KOA and TGF‐β groups compared to the Normal group, whereas the expression was significantly decreased in the TGF‐β inhibition group.

### 3.2 KOA synovial fibrosis initiates Netrin‐1 induction of sensory nerve germination

**FIGURE 2:**
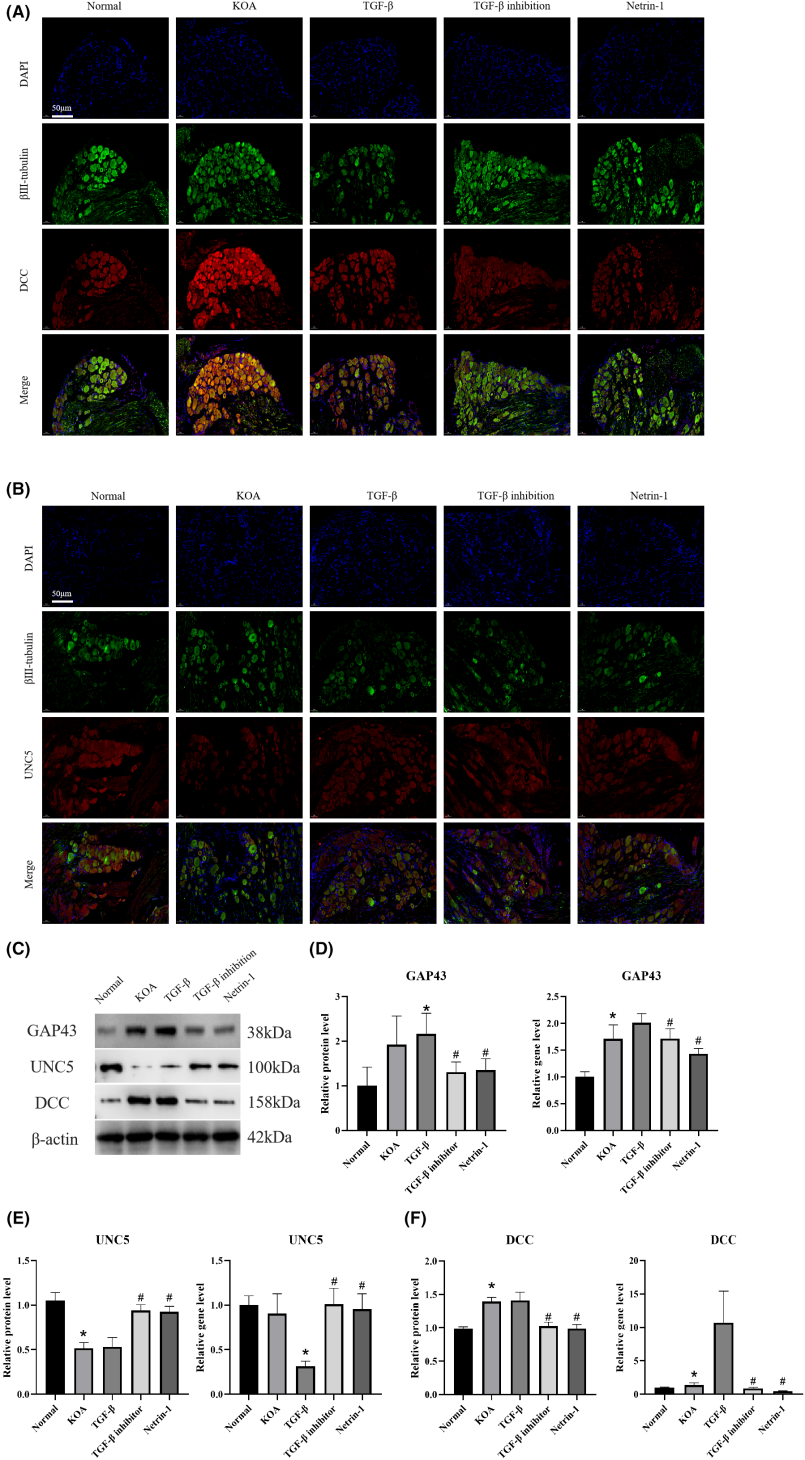
KOA synovial fibrosis initiates Netrin‐1 induction of sensory nerve germination. (A) Each group of DRG tissues was immunofluorescently stained using βIII‐Tubulin + DCC + DAPI at 200× scale bar = 50 μm to observe the expression of DRG markers and DCC, an indicator of nerve sprouting. (B) Each group of DRG tissues was immunofluorescently stained using βIII‐Tubulin + UNC5 + DAPI, 200× scale bar = 50 μm, to observe the expression of DRG markers and UNC5, an indicator of nerve sprouting. (C–E) WB and PCR were performed to detect the protein and gene expression of GAP43, DCC and UNC5, the neural sprouting‐related indicators in DRG tissues of each group of rats. (*p < 0.05, ^#^ *p* < 0.05).

In order to investigate the mechanism of KOA synovial fibrosis‐initiated Netrin‐1‐induced sensory nerve sprouting, DRG tissues from Normal, KOA, TGF‐β, TGF‐β inhibition and Netrin‐1 groups were subjected to βIII‐Tubulin+DCC+DAPI, βIII‐Tubulin+UNC5+DAPI immunofluorescence staining, and WB and qPCR were applied to detect the protein and gene expression of DCC, UNC5 and GAP43 in DRG tissues. The results are shown in Figure 2A,B. Compared with the Normal group, the expression of the neural sprouting indicator DCC was significantly increased in the KOA and TGF‐β groups, while the expression of UNC5 was significantly decreased, both of which highly overlapped with the βIII‐Tubulin‐labelled neuronal region, while the expression of DCC was significantly decreased in the TGF‐β inhibition and Netrin‐1 groups. Figure 2C–F showed that the expression of proteins and genes of GAP43 and DCC were significantly increased in the KOA and TGF‐β groups compared to the Normal group, while the expression of UNC5 was significantly decreased in the TGF‐β inhibition and Netrin‐1 groups. In the TGF‐β inhibition group and the Netrin‐1 group, the expression of GAP43 and DCC was significantly reduced compared to the KOA group and TGF‐β, while the expression of UNC5 was significantly increased.

### 3.3 KOA synovial fibrosis initiates Netrin‐1‐induced sensory nerve sprouting exacerbating KOA peripheral pain sensitivity

**FIGURE 3:**
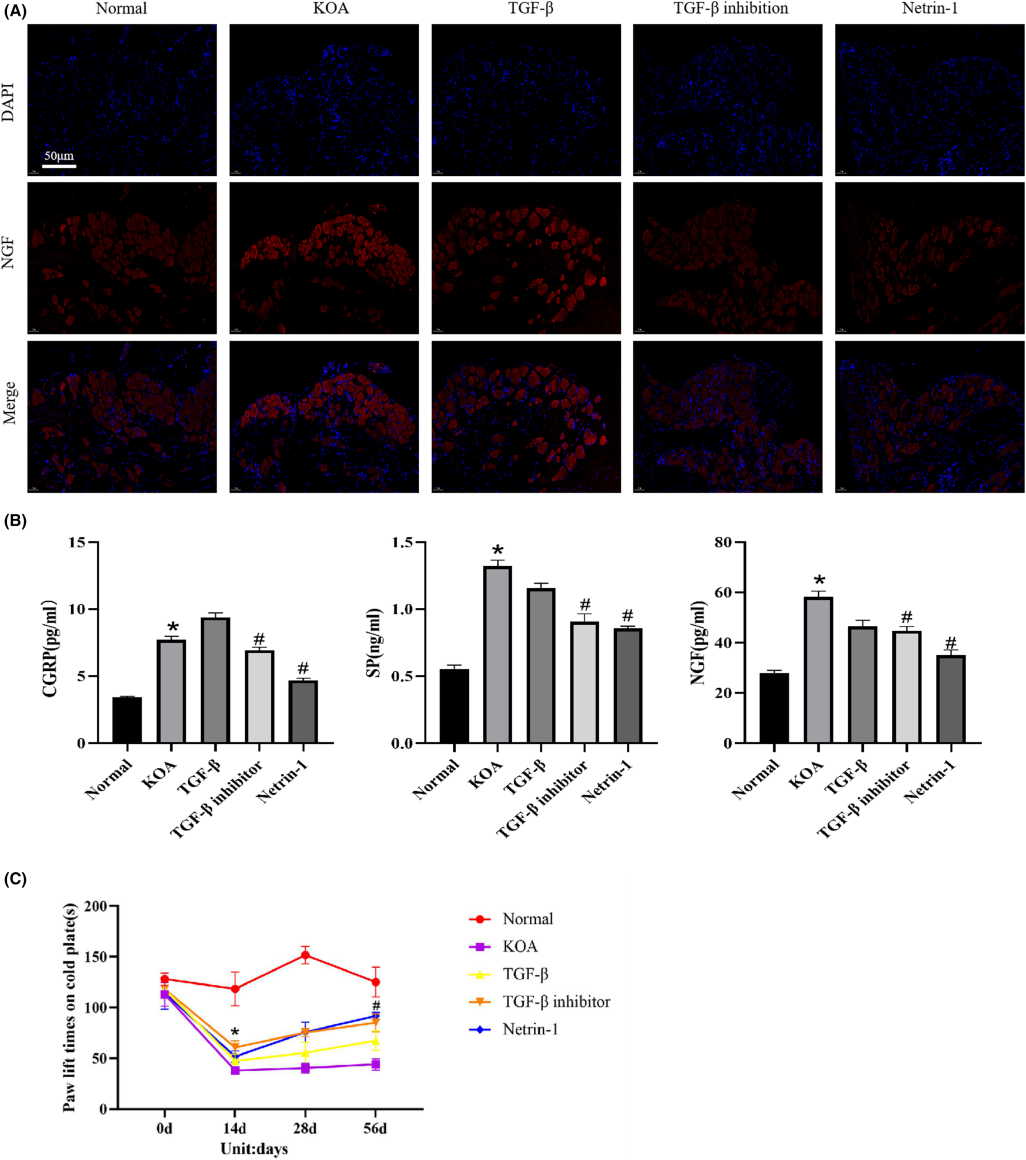
KOA synovial fibrosis initiates Netrin‐1‐induced sensory nerve sprouting exacerbating KOA peripheral pain sensitivity. (A) Immunofluorescence staining of DRG tissues of rats in each group with Netrin‐1 + NGF, 200× scale bar = 50 μm.(B) ELISA was performed on the sera of rats in each group to detect the expression of pain‐related factors CGRP, SP and NGF. (*p < 0.05, ^#^ *p* < 0.05) (C) Cold plate paw lift experiments were performed on each group of rats every 14 day and the paw lift time was recorded.(*p < 0.05, ^#^ *p* < 0.05).

To investigate the evidence of KOA synovial fibrosis‐initiating Netrin‐1‐induced sensory nerve sprouting exacerbating KOA peripheral pain sensitivity, ELISA was performed on the pain substances CGRP, SP and NGF in the blood of each group of rats. Cold pain sensitivity and mechanical pain sensitivity experiments were also performed on each group of rats to observe the pain‐related factor NGF in the DRG tissues of each group of rats under NGF immunofluorescence staining. The results are shown in Figure 3. As shown in Figure 3A, the expression of NGF in DRG tissues of KOA and TGF‐β groups was significantly increased compared to that of normal group, while the expression of NGF in TGF‐β inhibition group and Netrin‐1 group was significantly decreased. As shown in Figure 3B, the expression of pain‐related factors CGRP, SP and NGF in blood was significantly increased in the KOA and TGF‐β groups compared to the normal group, while the expression in the TGF‐β inhibition and Netrin‐1 groups was significantly decreased compared to the KOA group. In Figure 3C, at Day 14, the sensitivity to cold pain sensitization increased significantly and the paw lift time was significantly shorter in the KOA, TGF‐β, TGF‐β inhibition and Netrin‐1 groups; after the administration intervention, the paw lift time was prolonged in all groups at Day 28, while at Day 56 of the final administration, the paw lift time was significantly longer in the TGF‐β inhibition and Netrin‐1 groups compared to the KOA and TGF‐β groups. There was a statistically significant increase in cold pain sensitivity and a statistically significant decrease in paw lifting time in the KOA, TGF‐β, TGF‐β inhibition, and Netrin‐1 groups at Day 14 compared to Day 0, and a statistically significant increase in paw lifting time in the TGF‐β inhibition and Netrin‐1 groups at Day 56 compared to Day 14.

### 3.4 Vascular endothelial cell activation promotes the secretion of Netrin‐1

**FIGURE 4:**
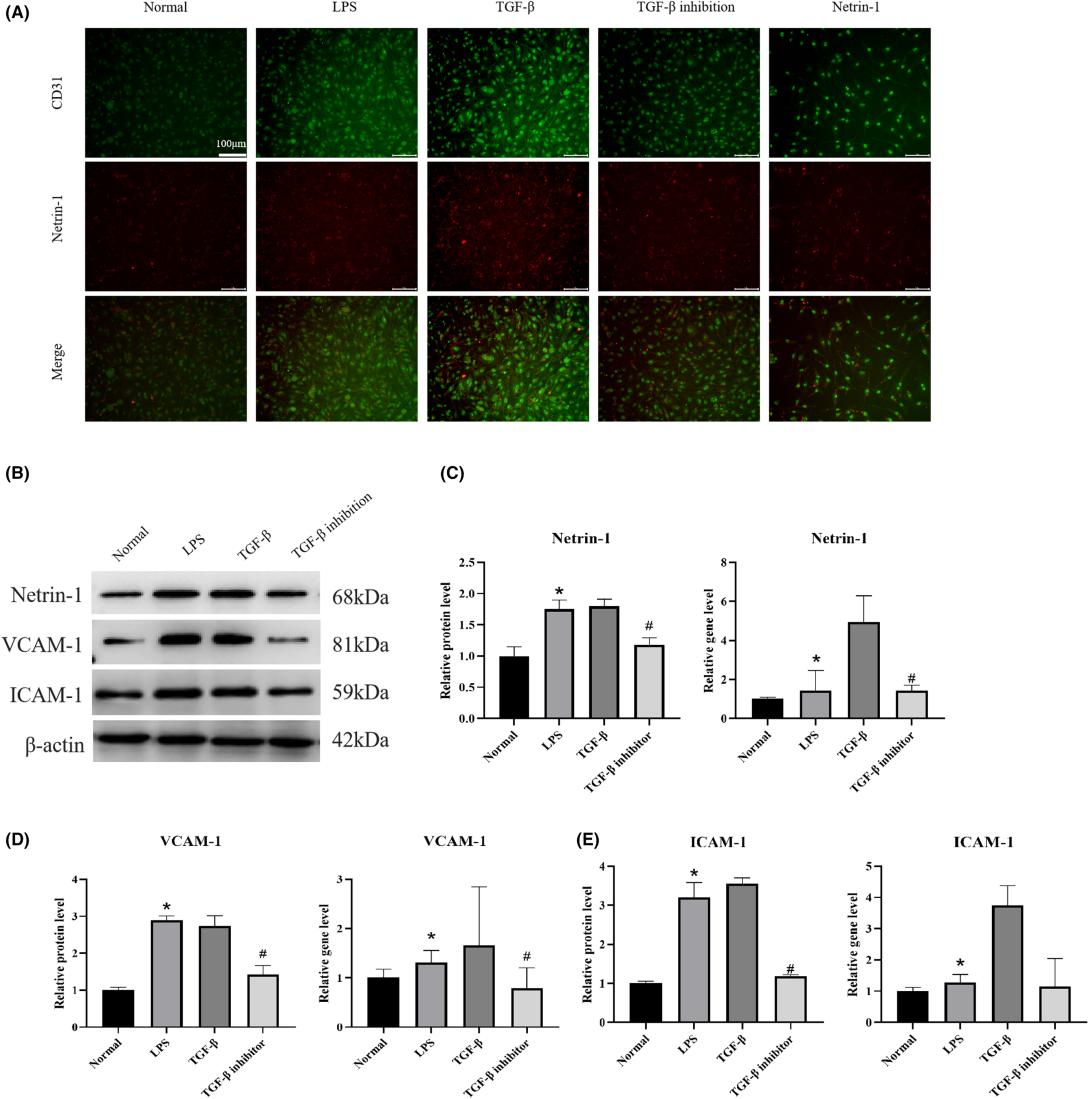
Vascular endothelial cell activation promotes the secretion of Netrin‐1. (A) Immunofluorescence staining of Netrin‐1 + CD31 was observed in HUVEC from each group after co‐culture, 100× scale bar = 100 μm, showing the expression of the vascular endothelial cell marker CD31 and the expression of Netrin‐1 in HUVEC. (B– E) WB and PCR were performed on each group of HUVEC to detect the protein and gene expression of Netrin‐1 and VCAM‐1 and ICAM‐1, indicators of vascular endothelial cell activation. (* p< 0.05, *^#^ p<* 0.05).

To investigate the specific mechanism of vascular endothelial cell activation to promote Netrin‐1 secretion, endothelial cells from normal, LPS, TGF‐β and TGF‐β inhibition groups were observed by immunofluorescence staining after co‐culture, and WB and qPCR was used to detect the protein and gene expression of Netrin‐1, VCAM1 and ICAM1 in endothelial cells. The results are shown in Figure 4. As shown in Figure 4A, the expression of Netrin‐1 and CD31 was significantly higher in the LPS and TGF‐β groups compared to the normal group with a high degree of overlap, while the expression in the TGF‐β inhibition group was significantly lower. In Figure 4B–E, Netrin‐1 and VCAM1, an indicator of endothelial cell activation, and ICAM1 protein and gene expression were significantly increased in the LPS and TGF‐β groups compared to the Normal group, and were more increased in the TGF‐β group compared to the LPS group, whereas they were significantly decreased in the TGF‐β inhibition group.

### 3.5 Activation of vascular endothelial cells by synovial fibrosis initiates Netrin‐1 to induce sensory nerve sprouting in vitro

**FIGURE 5:**
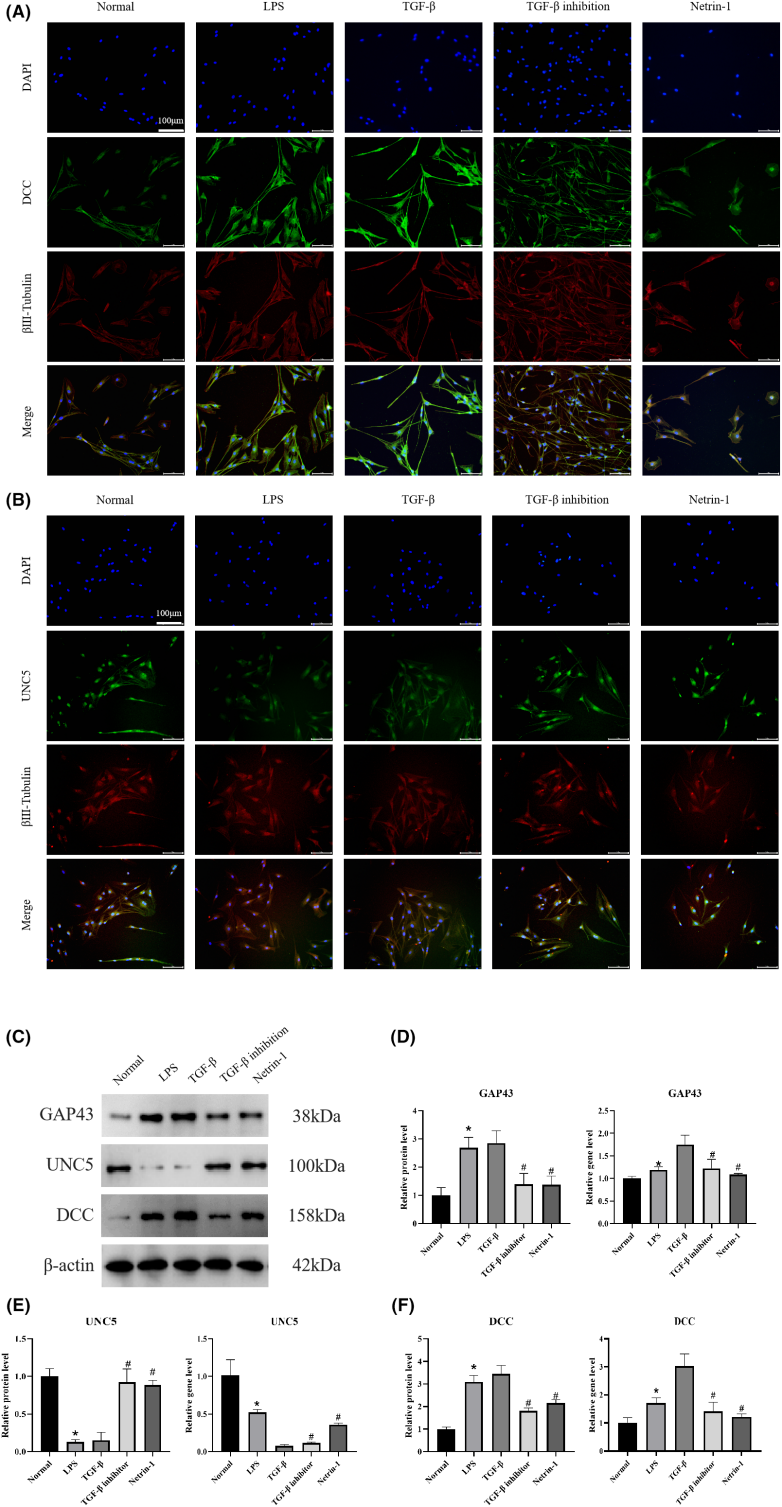
Activation of vascular endothelial cells by synovial fibrosis initiates Netrin‐1 to induce sensory nerve sprouting. (A) DRG neuronal cells after co‐culture in each group were stained using βIII‐Tubulin + DCC + DAPI immunofluorescence at 100× scale bar = 100 μm to observe the expression of DRG neuronal markers and DCC, an indicator of nerve sprouting. (B) DRG neuronal cells from each group after co‐culture were stained using βIII‐Tubulin + UNC5 + DAPI immunofluorescence, 100x scale bar = 100 μm, to observe the expression of DRG neuronal markers and the neural sprouting indicator UNC5. (C–F) WB and PCR were applied to detect the protein and gene expression of neuronal activation‐specific protein GAP43 and neural sprouting indicators DCC and UNC5 in DRG neuronal cells of each group. (*p < 0.05, ^#^ *p* < 0.05).

In order to investigate the specific mechanism of sensory nerve sprouting induced by Netrin‐1 secretion from synovial fibrosis‐activated vascular endothelial cells, the DRG cells in each group were stained with βIII‐Tubulin+DCC/UNC5 immunofluorescence, and the expression of DCC, UNC5 and GAP43 proteins and genes in DRG cells were detected by WB and qPCR. As shown in Figure 5A, the expression of βIII‐Tubulin and DCC in each group of cells was highly overlapping, with high expression of DCC in the LPS and TGF‐β groups compared to the normal group, and lower expression of DCC in the TGF‐β inhibition and Netrin‐1 groups compared to the LPS and TGF‐β groups. As shown in Figure 5B, UNC5 expression was reduced in the LPS and TGF‐β groups compared to the normal group, while UNC5 expression was increased in the TGF‐β inhibition and Netrin‐1 groups compared to the LPS and TGF‐β groups. As shown in Figure 5C–F, the protein and gene expression of DCC and GAP43 were significantly higher in the LPS and TGF‐β groups compared to the Normal group, while UNC5 was significantly lower. In the TGF‐β inhibition and Netrin‐1 groups, the protein and gene expression of DCC and GAP43 decreased significantly compared to the LPS group, while the expression of UNC5 increased significantly.

### 3.6 Synovial fibrosis initiates Netrin‐1‐induced sensory nerve sprouting exacerbating KOA peripheral pain sensitivity in vitro

**FIGURE 6:**
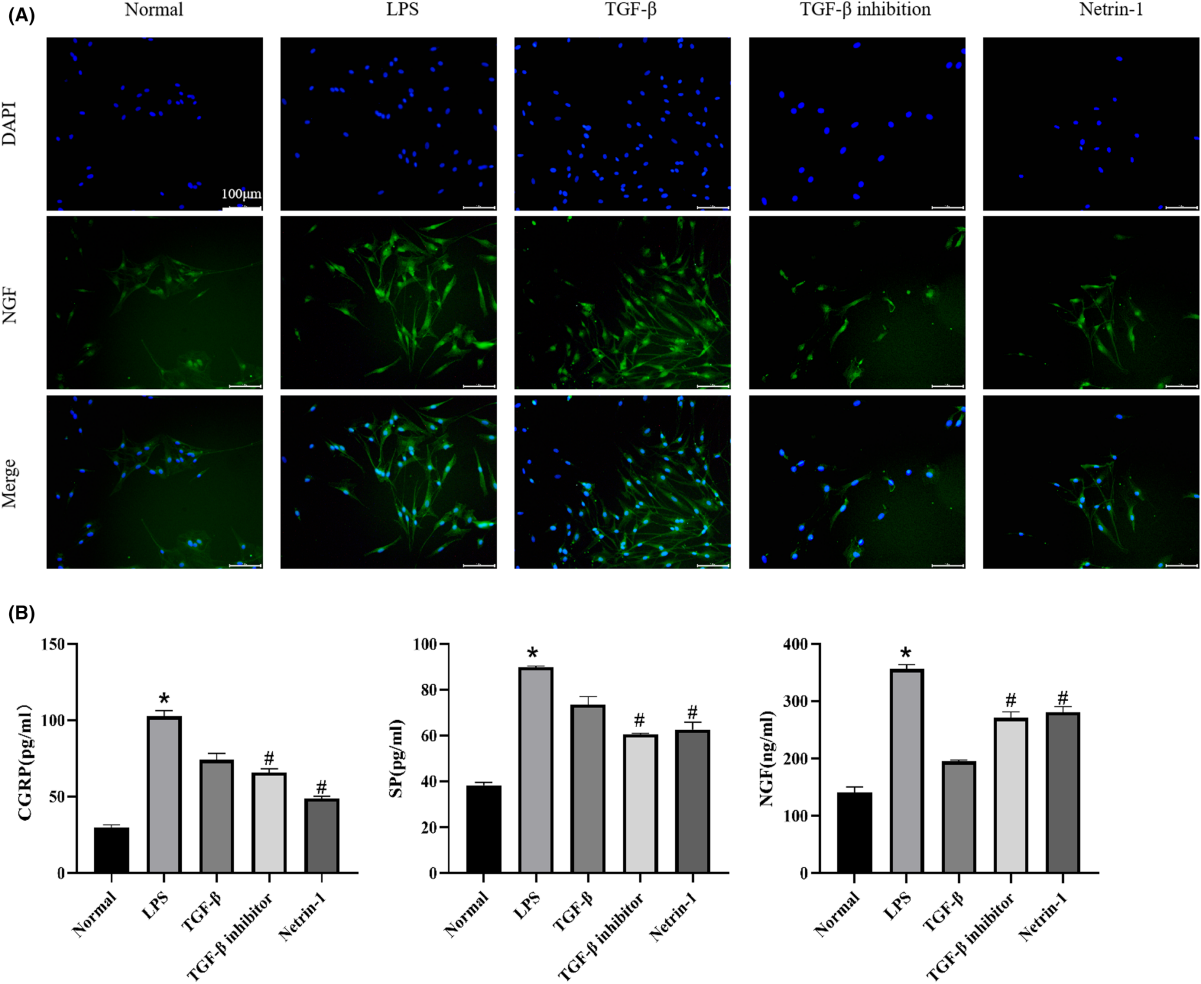
Synovial fibrosis initiates Netrin‐1‐induced sensory nerve sprouting exacerbating KOA peripheral pain sensitivity. (A) NFG immunofluorescence staining of DRG neuronal cells from each group after co‐culture to observe the expression of pain‐sensitive indicators. (B) The expression of pain‐sensitivity‐related factors CGRP, SP and NGF were detected by ELISA on the cell supernatants of each group after co‐culture. (*p < 0.05, ^#^ *p* < 0.05).

To investigate the effect mechanism of synovial fibrosis‐initiated Netrin‐1‐induced sensory nerve sprouting exacerbating peripheral nociception in KOA, ELISA was performed on the co‐culture supernatant of each group of cells to detect the expression of the nociceptive‐related factors CGRP, SP and NGF, and NGF immunofluorescence staining was also observed on DRG cells. The results are shown in Figure 6. In Figure 6A, the expression of NGF was significantly higher in the LPS and TGF‐β groups than in the normal group, while the expression of HGF was significantly lower in the TGF‐β inhibition and Netrin‐1 groups compared to the LPS and TGF‐β groups. In Figure 6B, the expression of pain‐related factors CGRP, SP and NGF was significantly higher in the LPS and TGF‐β groups compared to the normal group, while the expression of HGF was significantly lower in the TGF‐β inhibition and Netrin‐1 groups.

## 4 DISCUSSION

KOA is a chronic degenerative disease of the knee with pain as the primary clinical symptom and is widely present in the middle‐aged and elderly populations. Pain, as the main complaint of patients with KOA, has been one of the key propositions in KOA research; however, there are numerous factors that contribute to KOA pain, which can often be attributed to unvisualised intra‐articular soft tissue lesions and the inability to identify heterogeneous factors that contribute to pain sensitisation.^24^ Some studies have used magnetic resonance imaging (MRI) to make large observations and have shown that pain in KOA is associated with many structural factors within the joint, including the presence of bone marrow lesions (BML) and synovitis.^3^ There is also evidence that both peripheral and central nerve sensitisation may contribute to KOA pain during the development of KOA.^25^ Therefore, exploring the specific mechanisms that lead to KOA pain is important for the future targeted treatment of KOA pain.

Previous studies have demonstrated a close relationship between synovial fibrosis and KOA pain^5^; however, the synovial fibrosis environment activates vascular endothelial cells in synovial tissue, which promotes the formation of vascular opacities in the synovium, further leading to sensitization of peripheral pain, although the exact mechanism is unclear. In this regard, we conducted the present study to explore the potential mechanism of endothelial cell activation to promote Netrin‐1 secretion and affect peripheral pain. In vivo and in vitro experiments revealed that the expression of VCAM1 and ICAM1 proteins and genes, indicators of endothelial cell activation, was significantly upregulated in the setting of synovial fibrosis. We also found a significant increase in the expression of Netrin‐1 in synovial tissue in the KOA and TGF‐β groups, as well as in endothelial cells in the LPS and TGF‐β groups compared to the blank group. By performing multiplex immunofluorescence co‐localization of Netrin‐1 with CD31 in synovial tissue, we found that in the KOA and TGF‐β groups, the region of endothelial cell activation and the high overlap of the Netrin‐1‐expressing region and the enhanced signal indicated that the activation of endothelial cells in the synovial fibrosis environment promoted the secretion of Netrin‐1, which may be one of the important factors mediating nerve sprouting. In contrast to the KOA and TGF‐β groups, the TGF inhibitor and Netrin‐1 groups showed a low signal in the expressed region, suggesting that the activation of endothelial cells was significantly inhibited by the inhibitor intervention and that the secretion of Netrin‐1 was reduced, and this was confirmed in cellular experiments. The expression of DCC and GAP43 was found to be significantly up‐regulated in the KOA and TGF‐β groups compared to the blank group, while the expression of UNC5 expression was significantly down‐regulated in the KOA and TGF‐β groups compared to the blank group. In contrast, the expression of DCC and GAP43 was significantly down‐regulated in the TGF inhibitor and Netrin‐1 groups compared to the KOA group, whereas the expression of UNC5 was significantly up‐regulated. This further demonstrates that endothelial cell activation promotes Netrin‐1 secretion to mediate neural sprouting through the regulation of DCC/UNC5 repulsion and attraction.

Also, in the present study, we found that the activation of vascular endothelial cells to secrete Netrin‐1 mediated nerve sprouting under the influence of the synovial fibrosis environment was accompanied by a significant increase in the levels of pain‐related factors CGRP, SP and NGF in the serum as well as in the cell supernatant of the KOA, LPS and TGF‐β groups, which further indicated that nerve sprouting mediated the sensitization of peripheral pain by promoted the expression of pain factors, which further induced the production of KOA pain. In contrast, inhibition of synovial fibrosis and Netrin‐1 secretion resulted in significant reductions in CGRP, SP and NGF, indicating that peripheral pain sensitization was alleviated. In this regard, we also performed cold plate paw lifting experiments on each group of animals and found that the paw lifting time of rats in the TGF inhibitor and Netrin‐1 groups was significantly longer compared to the KOA and TGF‐β groups. In summary, activation of vascular endothelial cells by synovial fibrosis promotes Netrin‐1 secretion to mediate sensory nerve sprouting leading to peripheral pain sensitization in KOA probably through the attractive and repulsive effects of DCC/UNC5.

## AUTHOR CONTRIBUTIONS

**Zhenyuan Ma:** Writing – original draft (equal). **yibao wei:** Visualization (equal). **taiyang liao:** Validation (equal). **lishi jie:** Software (equal). **nan yang:** Supervision (equal). **likai yu:** Visualization (equal). **peimin Wang:** Writing – review and editing (equal).

## FUNDING INFORMATION

This work was supported by the National Natural Science Foundation of China (82074460, 82274545); Natural Science Foundation of Jiangsu Province (BK20201501); Jiangsu Province Postgraduate Training Innovation Project Grant (KYCX22_1943); Jiangsu Province Medical Key Discipline/Laboratory Construction Unit (js2252).

## CONFLICT OF INTEREST STATEMENT

All authors have reviewed this manuscript and indicated that there are no conflicts of interest regarding the content of this manuscript.

## CONSENT FOR PUBLICATION

All authors have provided consent for publication in the Journal of Chinese Medicine.

## Data Availability

All data contained in this document can be obtained from corresponding authors upon request. The original data is shared at the following link: https://www.jianguoyun.com/p/DU5HgCQQwrLMCxjpr4MFIAA.

## References

[1] Conaghan PG , Cook AD , Hamilton JA , Tak PP . Therapeutic options for targeting inflammatory osteoarthritis pain. Nat Rev Rheumatol. 2019;15:355‐363. 31068673 10.1038/s41584-019-0221-y

[2] Dieppe PA , Lohmander LS . Pathogenesis and management of pain in osteoarthritis. Lancet. 2005;365:965‐973. 15766999 10.1016/S0140-6736(05)71086-2

[3] O'Neill TW , Felson DT . Mechanisms of osteoarthritis (OA) pain. Curr Osteoporos Rep. 2018;16:611‐616. 30155845 10.1007/s11914-018-0477-1 PMC6153568

[4] Nanus DE , Badoume A , Wijesinghe SN , et al. Synovial tissue from sites of joint pain in knee osteoarthritis patients exhibits a differential phenotype with distinct fibroblast subsets. Ebiomedicine. 2021;72:103618. 34628351 10.1016/j.ebiom.2021.103618 PMC8511845

[5] Zhang L , Li M , Li X , et al. Characteristics of sensory innervation in synovium of rats within different knee osteoarthritis models and the correlation between synovial fibrosis and hyperalgesia. J Adv Res. 2022;35:141‐151. 35003798 10.1016/j.jare.2021.06.007 PMC8721247

[6] van der Kraan PM . The changing role of TGFbeta in healthy, ageing and osteoarthritic joints. Nat Rev Rheumatol. 2017;13:155‐163. 28148919 10.1038/nrrheum.2016.219

[7] Hulse RP . Role of VEGF‐A in chronic pain. Oncotarget. 2017;8:10775‐10776. 28099925 10.18632/oncotarget.14615 PMC5355219

[8] Mapp PI , Walsh DA . Mechanisms and targets of angiogenesis and nerve growth in osteoarthritis. Nat Rev Rheumatol. 2012;8:390‐398. 22641138 10.1038/nrrheum.2012.80

[9] Ashraf S , Walsh DA . Angiogenesis in osteoarthritis. Curr Opin Rheumatol. 2008;20:573‐580. 18698180 10.1097/BOR.0b013e3283103d12

[10] Bonnet CS , Walsh DA . Osteoarthritis, angiogenesis and inflammation. Rheumatology (Oxford). 2005;44:7‐16. 15292527 10.1093/rheumatology/keh344

[11] Das V , Kc R , Li X , et al. Blockade of vascular endothelial growth factor receptor‐1 (Flt‐1), reveals a novel analgesic for osteoarthritis‐induced joint pain. Gene Rep. 2018;11:94‐100. 30873504 10.1016/j.genrep.2018.03.008 PMC6411090

[12] Abe Y , Sugisaki K , Dannenberg AJ . Rabbit vascular endothelial adhesion molecules: ELAM‐1 is most elevated in acute inflammation, whereas VCAM‐1 and ICAM‐1 predominate in chronic inflammation. J Leukoc Biol. 1996;60:692‐703. 8975870 10.1002/jlb.60.6.692

[13] Finnerup NB , Kuner R , Jensen TS . Neuropathic pain: from mechanisms to treatment. Physiol Rev. 2021;101:259‐301. 32584191 10.1152/physrev.00045.2019

[14] Mantyh PW . Mechanisms that drive bone pain across the lifespan. Br J Clin Pharmacol. 2019;85:1103‐1113. 30357885 10.1111/bcp.13801 PMC6533434

[15] Gao R , Peng X , Perry C , et al. Macrophage‐derived netrin‐1 drives adrenergic nerve‐associated lung fibrosis. J Clin Invest. 2021;131:e136542. 33393489 10.1172/JCI136542 PMC7773383

[16] Schlegel M , Sharma M , Brown EJ , et al. Silencing myeloid Netrin‐1 induces inflammation resolution and plaque regression. Circ Res. 2021;129:530‐546. 34289717 10.1161/CIRCRESAHA.121.319313 PMC8529357

[17] Xia X , Hu Z , Wang S , Yin K . Netrin‐1: An emerging player in inflammatory diseases. Cytokine Growth Factor Rev. 2022;64:46‐56. 35082104 10.1016/j.cytogfr.2022.01.003

[18] Kennedy TE , Serafini T , de la Torre JR , Tessier‐Lavigne M . Netrins are diffusible chemotropic factors for commissural axons in the embryonic spinal cord. Cell. 1994;78:425‐435. 8062385 10.1016/0092-8674(94)90421-9

[19] Sharma M , Schlegel M , Brown EJ , et al. Netrin‐1 alters adipose tissue macrophage fate and function in obesity. Immunometabolism. 2019;1:e190010. 31428465 10.20900/immunometab20190010 PMC6699780

[20] Vosberg DE , Leyton M , Flores C . The Netrin‐1/DCC guidance system: dopamine pathway maturation and psychiatric disorders emerging in adolescence. Mol Psychiatry. 2020;25:297‐307. 31659271 10.1038/s41380-019-0561-7 PMC6974431

[21] Hashimoto Y , Toyama Y , Kusakari S , Nawa M , Matsuoka M . An Alzheimer disease‐linked rare mutation potentiates Netrin receptor uncoordinated‐5C‐induced signaling that merges with amyloid beta precursor protein signaling. J Biol Chem. 2016;291:12282‐12293. 27068745 10.1074/jbc.M115.698092 PMC4933276

[22] Li HP , Su W , Shu Y , et al. Electroacupuncture decreases Netrin‐1‐induced myelinated afferent fiber sprouting and neuropathic pain through mu‐opioid receptors. J Pain Res. 2019;12:1259‐1268. 31118749 10.2147/JPR.S191900 PMC6499485

[23] Zhu S , Zhu J , Zhen G , et al. Subchondral bone osteoclasts induce sensory innervation and osteoarthritis pain. J Clin Invest. 2019;129:1076‐1093. 30530994 10.1172/JCI121561 PMC6391093

[24] Vincent TL . Peripheral pain mechanisms in osteoarthritis. Pain. 2020;161(Suppl 1):S138‐S146. 33090747 10.1097/j.pain.0000000000001923 PMC7434216

[25] Katz JN , Arant KR , Loeser RF . Diagnosis and treatment of hip and knee osteoarthritis: a review. JAMA. 2021;325:568‐578. 33560326 10.1001/jama.2020.22171 PMC8225295

